# Exploring Eating Challenges and Food Selectivity for Latinx Children with and without Autism Spectrum Disorder Using Qualitative Visual Methodology: Implications for Oral Health

**DOI:** 10.3390/ijerph18073751

**Published:** 2021-04-03

**Authors:** Lucía I. Floríndez, Daniella C. Floríndez, Mia E. Price, Francesca M. Floríndez, Dominique H. Como, Jose C. Polido, Lourdes Baezconde-Garbanati, Elizabeth Pyatak, Sharon A. Cermak

**Affiliations:** 1 Department of Nursing Research, Cedars Sinai Medical Center, Los Angeles, CA 90048, USA; 2 SOS Mentor, Los Angeles, CA 90089, USA; daniellaflorindez@gmail.com; 3 USC Mrs. T.H. Chan Division of Occupational Science and Occupational Therapy in the Herman Ostrow School of Dentistry, University of Southern California, Los Angeles, CA 90089, USA; miaprice@usc.edu (M.E.P.); dcomo@usc.edu (D.H.C.); Beth.pyatak@usc.edu (E.P.); cermak@chan.usc.edu (S.A.C.); 4 Willamette Academy, Willamette University, Salem, OR 97301, USA; fmflorindez@willamette.edu; 5 Division of Dentistry, Children’s Hospital Los Angeles, Los Angeles, CA 90027, USA; jpolido@chla.usc.edu; 6 Keck School of Medicine, University of Southern California, Los Angeles, CA 90089, USA; baezcond@usc.edu

**Keywords:** Autism Spectrum Disorder, children, families, health disparities, Latinxs, nutrition, oral care, photo elicitation, qualitative

## Abstract

Diet and food choices significantly impact teeth, including enamel quality and development of dental caries. However, studies focusing on diet and its relation to oral care in Latinx children with and without Autism Spectrum Disorders (ASD) have been minimally addressed in research. This qualitative study used an inclusive visual methodology to explore what Latinx caregivers learned about their child’s diet preferences and food routines in relation to their oral health. As a secondary aim, the study sought to explore whether notable differences in diet emerged between Latinx children with and without ASD. Participants were 32 Latinx caregivers from 18 families with children with and without Autism (*n* = 8 with a typically developing child and *n* = 10 with a child with ASD) who completed a food journal activity and photo elicitation interview. Interviews were thematically coded for themes pertaining to parents’ perceptions of their child’s diet and oral health. Findings of this study indicate that the process of taking photos helped Latinx caregivers to better situate the barriers and behaviors influencing everyday food routines in their children within the context of relating to their overall oral health. Via their active participation in the research process, parents were empowered to note strategies they could employ that would directly impact their child’s oral health outcomes, such as reducing juice intake and monitoring sugar consumption. Therefore, visual research methodologies are an important strategy for researchers to consider in order to empower participants to be part of the research process and part of the outcomes, and to offer better understanding of the lived experience of populations underrepresented in the literature, such as Latinx children with and without ASD and their families.

## 1. Introduction

Studies focusing on diet and its relation to oral care in Latinx (*Throughout this article, we use Latinx, a gender neutral term to indicate someone of Latino/a ethnicity*) children with and without Autism Spectrum Disorders (ASD) have been minimally addressed in research. However, this is a key area to focus investigations, as Latinxs [1,2,3] and children with ASD [4,5] disproportionately experience oral health problems when compared to the general population, and there are strong ties between diet and dental health [6]. Diet and food choices significantly impact teeth, including enamel quality and development of dental caries [7]. A diet high in nutrients like vitamins A, C, and E, and calcium aids in promoting healthy dentition [6]. However, few children and adolescents meet the current recommendations concerning optimal nutrition guidelines [8].

When race/ethnicity is considered, Latinxs in the US consume a less healthful diet compared to other ethnic and racial groups in the country [9]. Factors that contribute to diet for Latinxs include poverty and cost of healthy food [10], embedded cultural factors [11], limited education [12], and reduced health literacy [1], and food insecurity [13]. Research on early childhood caries risk among the Latinx population explains some of the risk factors leading to poor oral health outcomes. Firstly, it is reported that Latinx children have a higher consumption of soft drinks and lower intake of fruits [14], decreased vegetable consumption and increased soda and chip consumption [15], and reduced access to fruits and vegetables [16] compared to their non-Latinx White counterparts. Additionally, higher levels of acculturation and assimilation into American culture correlate with decreased dietary quality among Latinx children [9,17] and higher body mass index (BMI) [18], plus higher rates of obesity and diabetes [19]. Latinxs who have lived in the US longer consume less fruit, rice, and beans and more sugar and sugar-sweetened beverages, fast food, snacks, and food with added fat than Latinxs outside of the United States [20], which also increases their likelihood of having caries [21].

Research has also shown that children with ASD, which has risen in prevalence to occur in 1 in 54 children [22], often exhibit challenging eating behaviors that can lead to later health consequences. For example, while not specific to Latinxs, children with ASD have been noted to have difficulties with food preferences and mealtime routines due, in part, to their sensory sensitivities [23,24,25]. Indeed, food selectivity, defined as accepting a limited list of foods into regular diet [23], is estimated to be present in almost 80% of children with ASD [24], resulting in restrictive diets and food habits. Food selectivity is also associated with nutritional inadequacies [23] and poorer oral health outcomes, including increased prevalence of malocclusion, dental calculus, and dental plaque levels [26]. Additionally, the incidence of obesity in children with ASD is 30.4% compared with 23.6% in typically developing (TD) children [27]. Moreover, a higher prevalence of oral caries has been reported in children with ASD [28], as well as well-documented issues surrounding disparities in obtaining adequate oral care [29,30], finding a dentist willing to provide care [31,32], and performing oral care routines [5,33].

Given the oral health challenges related to diet and feeding practices, more research is needed exploring the perspectives of these populations that are underrepresented in current literature. While exploring families’ perspectives about their diet and oral health, a participative methodology can be helpful to be inclusive to the parent, identifying them as a researcher and key-informant, and including them in the process of *doing* the research. Photo journal projects often include photo elicitation interviews (PEI), which use participant-taken photographs to guide the interview questions while learning from the perspective of the participant. Wang [34] suggested an acronym, SHOWeD, to summarize the elements of a dialogue during a photo elicitation interview: What do you See here? What is really Happening? How does this relate to Our lives? Why does this situation exist? What can we Do about it?

Visual methodologies (VM), like photovoice and photo journals, have been used to explore Latinx/a children’s perspectives of health care [35], among Latinx caregivers exploring navigating limited food environments [36], and within the special healthcare needs/disability community [37], including with parents with children with ASD [38]. Given the inclusive and participatory nature of a VM, it was determined to be an appropriate method to utilize in a research study with an underserved and under researched population. While not constructed as a true Photovoice project, this study was informed by the principles of using an inclusive, photo-based visual research methodology to study the lived experiences of Latinx families. Thus, the purpose of this study was to explore how the process of visual food journaling provided an opportunity for Latinx families who had children with and without ASD in Los Angeles, California to better understand their diet and food choices in relation to their oral health.

## 2. Materials and Methods

### 2.1. Research Design

This article presents a sub corpus of findings from a study of oral health care knowledge, attitudes, and practices of Latinx families with children with and without Autism [39,40]. The present study used a qualitative approach to analyze PEIs and visual food journals with Latinx parents/caregivers to identify what these parents learned about their children’s nutrition and oral care from the process of taking and discussing photos. Ethical approval was granted by the University of Southern California Institutional Review Board (HS-16-00921).

### 2.2. Participants

Participants were 32 parents/caregivers from 18 families (10 with a child with ASD and eight with a typically developing (TD), neurotypical child). Participants were purposefully recruited to meet eligibility criteria through word of mouth, via social media, as well as through partnerships with community centers. Eligibility criteria for participants were (a) self-identify as Latinx; (b) able to speak, read, and write either English or Spanish; (c) parent of a child between ages 6–12 who is TD OR is diagnosed with ASD by a licensed professional, and (d) use dental services in the Los Angeles, California area. All study documents were professionally translated into Spanish, including consent forms, recruitment materials, and interview guides. All participants signed consent forms prior to being interviewed.

### 2.3. Procedures

Three bilingual study team members conducted semi-structured, audio-recorded PEIs with *n* = 18 Latinx primary caregivers in English or Spanish, based on participant preference. PEIs lasted 45–190 min, and all families received compensation for their time.

The visual food journals were completed by the participants prior to the PEI. To complete the journals, participants were instructed to take pictures of their child’s meals for 3 days of the week, to include both weekdays and weekends, and show the contents of the meal. Each family was empowered to choose the layout and visual presentation of each photo, so long as they included photos pertinent to diet and food choices. Participants used their own cell phones to document the photos; in cases where participants did not have the equipment to photograph their child’s food, the research team provided them with supplies to be able to complete the activity. Following the photo data collection period, which varied from family to family but averaged about three weeks, the PEI took place at the participant’s home. Informed by elements from the SHOWeD prompts [34], during each semi-structured interview the participant was asked about their daily dietary routines and the contents of the photographs. In addition to the SHOWeD prompts, supplemental interview questions are included in Table 1. This interview and engagement with participants were also an opportunity to engage in member checking, validating study findings with the participants to ensure accuracy of data [41].

### 2.4. Data Analysis

All interviews were transcribed verbatim, and a professional service was used to translate Spanish language transcripts into English for analysis. Photos from food journals were printed and catalogued by participant. The research team also took inventory of all of the foods that appeared in the photos to note type of food, whether the food was nutrient dense or not, and the frequency in which the food appeared across pictures in the sample. The food inventory list generated from the photos was cross checked with the interview transcript to ensure accuracy of food item recording. For transcript analysis, four research team members independently reviewed the interview transcripts several times to become familiar with the data. Using a thematic analysis approach [42] and informed by the themes discussed during the interview with the participants themselves, a preliminary set of codes was developed to identify significant statements in the text with reference to perceptions about diet and oral health in the families. The entire research team met to discuss and refine codes to create the data sub corpus for this analysis. Several strategies were used to ensure rigor and trustworthiness in the data. Triangulation was performed by including the perspectives of the entire research team when analyzing data, as well as drawing inferences from different data sources, such as the photos, interviews, and observations. As a form of member checking, themes and supporting quotes were presented to a selection of participating families who had expressed interest in further contributing to the research process (four families with neurotypical children and four families with children with ASD) to get their feedback about content and organization. Lastly, the research team maximized reflexivity by discussing their personal biases as they emerged, and documenting their thoughts in field notes and meeting minutes to reflect on how the biases may be shaping the research process. As all parties agreed on the code definitions, research team members proceeded to fully coding the transcripts using QSR’s NVivo 11 software (QSR International Pty Ltd. (2018) NVivo (Version 11), Melbourne, Australia) based on this refined coding scheme.

## 3. Results

### 3.1. Sample Characteristics

In this study, visual food journals and subsequent PEIs were central to framing how food choices of Latinx children relate to diet and oral health. Demographic information was self-reported from participants and is detailed in Table 2. In order to maintain anonymity, any identifying information was altered and pseudonyms were assigned to all participants. Of the 18 participating families, 10 had a child with ASD (nine male and one female), and eight had a neurotypical child (two male and six female). We collected no questionnaires about intellectual functioning or specific sensory sensitivities of the children in the study; all information about child characteristics is based on parent report and participant observations. Firstly, all of the child participants were able to eat autonomously, and had at least moderate language skills to communicate about their food preferences. Of the children with ASD, all parents noted that their children had certain food preferences that made meal time and eating more difficult, that introducing new foods was challenging, and there were some things that their children would not ever eat. Similarly, parents of the TD children did sometimes describe their children as “picky”, but never mentioned any specific food aversions in their children.

The families with children with ASD primarily self-identified as Mexican-American (*n* = 6); of the remaining families, *n* = 3 identified as Central American and *n* = 1 identified as South American. The families with TD children primarily self-identified as Central American (*n* = 4); of the remaining families, *n* = 3 identified as Mexican-American and *n* = 1 identified as South American. Caregivers provided a total of 162 photos for review during the photo elicitation interviews, ranging from 5 to 27 photographs per family.

### 3.2. Findings

From the data, the themes that arose were: “Maybe I don’t do as well as I thought I did” (VM as opportunity to identify areas where the parent wants to improve eating patterns); “He is actually pickier than what I thought” (VM as opportunity to learn more about children’s eating patterns); “Food became fun” (VM as means of creating change and encouraging children to try new foods); “At home, we eat better” (VM as opportunity to highlight strengths of family’s eating patterns); and “Sugar is the devil” (VM as highlighting the negative impact of consuming too much sugar). Relevant information from the food inventory list created by the research team appears in Section 3.2.5.

#### 3.2.1. “*Maybe I Don’t Do as well as I Thought I Did*” (VM as Opportunity to Identify Areas Where the Parent Wants to Improve Eating Patterns)

This theme focused on visual methodology as opportunity for parents to identify areas where they want to improve eating patterns. In some cases, parents looked at nutrition labels and realized the dietary content of the food they gave their child. Other times, parents were literally faced with their choices when reviewing the photos of what they allowed their children to eat. By analyzing the eating patterns of their children once time had passed and they were no longer immersed in the activity, parents could reflect on their feelings about where they could make changes in the future. For example, when asked to talk about her thoughts on the food journaling project, Sara, mother of 8-year-old Marco with ASD, elaborated on her personal takeaways, which included lamenting the fact that Marco ate sugary food that could impact his dental health:

“*I thought in my mind that I did a really good job of like watching sugar content and making sure that they’re eating healthy but then when I started taking pictures and looking at labels I was like ‘oh boy, oh ok, maybe I don’t do as well as I thought I did.’ And it was good though, it was a good, it was a good, kind of helping me to realize like what I really do feed my kids. I don’t think I’ve changed it as of yet, but it’s something that I’m thinking about now. Like okay, what are some replacements? What are some options? Where can I cut back on? … Like it definitely made me look again at our diet and what we’re feeding our kids and stuff because looking at them labels, man. The cereal he eats is basically all sugar. And sugar pretty much rots your teeth. That wasn’t a happy thing to realize, that I pretty much give my kid things that will potentially damage his teeth.*”

Violeta noted that her 12-year-old son Justin, who has ASD, “asks more questions than what I notice”, and lamented the fact that sometimes she was too busy to include her son in the food preparation process despite his showing interest in being involved. She continued:

“*You know sometimes, when you’re in the moment [making food for the family], you just, you know, you just answer or ignore him sometimes, and I’ve found that he was very curious … he was asking me more, ‘Can I help? Can I help?’ And I should have been like, ‘okay, yeah sure.’ But sometimes you get busy. And I need to include him while he still asks me about it.*”

Here, she was able to critically reflect on the fact that she should capitalize on the fact that her son wants to be involved in the food process while he is still at an age where he wants to be engaged in learning.

For Celia, she lamented the high costs of fresh fruit and vegetables, and regretted that she only knew how to prepare food in the ways she learned from her own mother, and not in ways that were appetizing enough to entice her neurotypical daughter, 7-year-old Ana Sofia, to actually eat any vegetables that she did manage to purchase. Celia’s predicament was that she knew that vegetables were part of a healthy and nutritious diet, but did not know how to incorporate them into Ana Sofia’s diet due to costs and food preparation limitations. Celia described: “*It costs me more to get them vegetables. They are so expensive. But they are good for you, and make your body and your teeth healthy. And I do the most I can, but I do have to learn to prepare the vegetables in a way they like, and not like I already know how to*.” Celia further expanded on the cost of fresh fruit being an issue because her all four of children loved certain fruits, and she felt badly when she could not afford to provide them for her kids. She also noted that the fruit that she found most affordable, apples, was a “good for you” food to include in her child’s diet:

“*They love fruit … they love oranges, plums, mangoes, grapes, strawberries. All kinds. But they like fruit so much that it can get expensive to buy so much for them. I wish I could buy them fruit all the time. Usually we have just what I can afford, like apples. But don’t they say that apples are good for you?*”

Here, Celia is describing a crucial predicament pertaining to the high cost of healthy eating, especially as it involves buying nutrient dense food for multiple family members or growing children with hearty appetites. Valentina, mother of 11-year-old neurotypical Ximena, also acknowledged that healthy food often comes with a high price tag, or is less readily available, and wished she had more resources to spend on the expensive food. She described a picture of a naturally squeezed juice with a story:

“*This picture is a juice with kale, spinach and lemon. We were in Whole Foods yesterday making our weekly purchases, and it’s so lovely that they make fresh juice there, so we paused to have a juice. [The juice] has only 170 calories, but they are all good calories because the juice is pure vegetables. They even let us try to the juice to see if it was too acidic, because they had put a good amount of lemon in there. How nice of them, right? But the juice is expensive. Thirty dollars for the 3 of us to have a fresh juice. And the thing about Starbucks is they are cheap, and they make things in whatever way they make them, with all the calories, and they just give you the drink and say ‘here it is, take it’. But Starbucks is everywhere and it is popular, and Whole Foods is expensive, so we go there less.*”

Cost of food also impacted Isabella’s meals for her son, Zach, age 8 with ASD. Isabella said:

“*Changing diet is kind of hard as well, because sometimes you don’t have the money for it … even though you’re supposed to eat one food or another, how do you afford it? … Especially salmon. Like, that’s their favorite, and just a piece of salmon is, like, $23, $24. And they don’t just eat that little portion, it has to be a big portion. But if I cannot buy salmon, then I buy a cheaper fish, so they can still get the nutrition.*”

Araceli wanted to be more involved in her 7-year-old neurotypical daughter Julia’s eating process by determining more of Julia’s meal contents, but did not know how to insert herself into Julia’s routine more. Araceli said: “*Julia is so independent. She eats what she wants and grabs it for herself from the refri[gerator], does it all by herself. I wish I helped her more. But, at least she eats healthy. Her favorite food is vegetables. Sometimes I walk in and just find her eating carrots.”*

Notably, this comment about independently preparing and consuming food was not one shared by parents of children with ASD. For example, Valeria wanted to encourage her 11-year-old son with ASD, Juan, to eat more nutritious food, and wished she did not give in to his demands so frequently. In reviewing the pictures, Valeria commented:

“*I noticed not enough veggies. He’s still very like against veggies or even tasting it, the textures or the taste of the vegetables. He just won’t have it … An example is going to the mall, because one day we did go to the mall and he had Hotdog on a Stick and I’m just like ‘Oh my god, I can’t believe I let him have that.’ He’s such a picky eater but he knows where to go to if we go to the mall. That made me think like ‘okay what should I change? Should I not give in to him when he wants to eat all these things?’ Especially these things that are just not even remotely good for him.*”

#### 3.2.2. “*He Is Actually Pickier Than What I Thought*” (VM as Opportunity to Learn More about Children’s Eating Patterns)

This theme pertains to VM as opportunity to learn more about children’s eating patterns. Here, parents discussed their observations of their children’s dietary habits, and how the photo taking process expanded what they knew about their child’s preferences and routines. With the camera in their hand, parents took on the role of active observers, and were able to discuss the particularities of their child’s food preferences with the research team. These comments were invaluable to understanding more about food selectivity in the child participants. Actually, several parents of both children with ASD and TD children noted how selective their children were about what they consumed. Violeta (parent) was asked to describe anything else she wanted to note about the process of taking pictures of food, and noted her realization about her 12-year-old son with ASD, Justin. Violeta said, “*He is actually pickier than what I thought. You know, like I was like ‘oh I didn’t know he wasn’t crazy about this food’*.” Similarly, parent Carla described her neurotypical 8-year-old daughter Samantha by saying “*she pretty much eats the same thing. I saw so many blueberries in all the pictures, she eats them all the time*.”

Another parent, Camila, with a 12-year-old son with ASD, talked about one photo she took of son Stefan’s food by saying:

“*This salad includes lettuce, spinach, and carrots … He’s very picky. He has gotten pickier. Especially with vegetables. The most vegetables that he eats is lettuce, spinach, and carrots. That’s the only three vegetables that he eats … And carrots. Uh-huh. He’s very, very picky. He don’t [sic] like green beans. He don’t like bell pepper. He don’t like broccoli or squash. Even cucumber. He don’t like cucumber. And tomato. He don’t like the tomato. I didn’t know about the tomato.*”

Celia also described how particular her neurotypical 7-year-old daughter Ana Sofia was when talking about a picture of food from McDonald’s.

“*Well, this is a picture from McDonald’s. They always want McDonalds, and my husband says, ‘no, that place is not healthy. A better idea is to go to Subway or something. And Ana Sofia will say ‘no, I don’t like such and such place’ and if we go to Subway, she gets a ham sandwich and then has us take off all the vegetables … and we go out from time to time, when we can afford it, but she always wants McDonald’s … and at McDonald’s she only eats chicken nuggets, and even then, it’s only so she can get the toy from the happy meal. Or if we get Panda Express, she only eats chow mein. That’s it.*”

Valentina commented on her 11-year-old neurotypical daughter Ximena’s food preferences, and noted the nutritional components of each option, including describing sugar content:

“*… There are two things she really loves to eat, and I learned that it depends on where we are, what she eats. One is Starbucks, and the other is natural juices. From Starbucks, we already know the drink is really delicious, but it has too much sugar, too many processed elements and ingredients that are bad for your teeth and for your health. And the other option she loves is fresh natural juice.*”

From these comments, it became apparent that food selectivity and picky eating was commonly mentioned across the sample of children, and not unique to only the children with ASD.

#### 3.2.3. “*At Home, We Eat Better*” (VM as Opportunity to Highlight Strengths of Family’s Eating Patterns)

All content in this theme pertains to VM as opportunity to highlight strengths of family’s eating patterns. Adriana, mother of 10-year-old neurotypical daughter Cristina, jokingly declared: “*obviously we already kind of knew we ate a lot of frijoles and that most of the time, we eat rice. But that’s better than like, dessert all the time*.” She also went on to describe how she was pleasantly surprised with how they had reduced the amount of times they ate out at fast food “unhealthy” restaurants on a weekly basis, and attributed this habit change to the fact that her mother-in-law was visiting from Latin America and had largely taken over food preparation activities for the family. Adriana was grateful for the support offered by her family member in preparing nutritious and healthy meals, and said:

“*I just realized that actually when I was taking the pictures and thinking about what is the restaurant you frequently visit, I was asking my mother-in-law, and I go, ‘When was the last time we went out to eat? Cause like I can’t remember.’ [The food journaling process] asking me to document these things made me see how much my mother-in-law cooks for us now. And then, oh yeah, I did notice the changes that we were eating more here at home, and it’s food that is better for us, you know, the home-made arroz y frijoles [rice and beans] and not the greasy stuff from fast food. So at home, we eat better, you know? And maybe just once, maybe twice in a month is the most I think we eat out now that she [mother-in-law] is here.*”

Other parents were also pleased with the outcomes of reviewing the food photos during the PEI. Armando, father of 7-year-old Perlita with ASD, described how they felt all the pictures were accurate to their real life daily experiences. Armando noted:

“*Well, we are vegetarian, and so we eat a base of vegetables and lots of legumes. So that is what you see in every photo, because that is what our life is like. We also eat lots of dairy products like cheese and milk, and also eggs. Also we eat a lot of seeds, nuts, and almonds. We try to eat healthy, live healthy. We even brush our teeth after every meal. And we think it shows in our pictures.*”

#### 3.2.4. “*Sugar Is the Devil*” (VM as Highlighting the Negative Impact of Consuming too much Sugar on Oral Health)

This theme surrounds how VM highlighted how parents realized the negative impact of consuming too much sugar, and how it could affect their children’s oral health status. Parents also discussed their strategies for reducing sugar consumption in their children. Mia, mother of 11-year-old neurotypical Xochitl, described sugar by saying: “it is the worst thing for her teeth. The very worst thing. So that’s why I barely let her drink soda. Because of all the sugar.” Sara, mother of 8-year-old Marco with ASD, noted that:

“*We do a lot of peanut butter and jellies, he eats a lot of fruit. I didn’t think I did a lot of snacks, but like watching and like looking at their actual food journal and having to take pictures of everything else, I was like, mm, maybe I am offering too many snacks, maybe I could offer him more veggies, or you know, less sugary things instead. Because sugar, man, it gets you.*”

Another parent of a neurotypical child, Celia, commented about a strategy that she used to avoid sugar late at night, while also being cognizant of her food budget:

“*They love fruit and juice. But it gets so expensive if they drink it all the time, more than I can afford. And juice is too sugary. So after 4 pm, no more fruit. And they have to have water with dinner. Those are the rules.*”

During her photo elicitation interview, Jennifer elaborated on her goals for her 7-year-old son with ASD, Luca, and her candid thoughts on sugar:

“*I’d just like him to have a balanced diet. So good amounts of sugar and good amounts of carbohydrates, and I would rather have him learn how to properly learn what’s good for him, so-because I think all of our parents say, oh, eat fruits and vegetables. It’s so good for you. And then you realize when you’re older and you’re, like, oh, my gosh, even fruit and vegetables even have a lot of sugar in them, and sugar is the devil …*”

After looking at a picture of maple syrup that she uses on Luca’s breakfast pancakes, Jennifer explained more about the pitfalls of sugar:

“*Oh my God this syrup has 48 g of sugar in it! Do you know your child is only supposed to have, like, six [grams of sugar] or something? … and you know apple juice has, like, 46 g of sugar in it. And I’m, like, ‘I know we water Luca’s sugar down’, we water down his juices when we give it to him … but still, like, that’s not counting his peanut butter, that’s not counting his bread, that’s not counting his syrup, that’s not counting like, all these other things, and I’m just like, ‘oh, dear God.’ And from what I understand, which is, you know, I feel like sugar just sticks to your gums and your teeth and it just resides there, and then bacteria says, ‘oh, yum, sugar. Well, let’s dig in,’ and then starts eating away at your body basically, starting with your teeth.*”

#### 3.2.5. “*Food Became Fun*” (VM as Means of Creating Change and Encouraging Children with ASD to Try New Foods)

This theme pertains specifically to comments made by parents of children with ASD, about using the process of VM as a means of creating change and encouraging their children to try new foods. Some parents noted how the photo journaling process facilitated meal time with their children who were normally much more selective about their food choices. Parent Paola noted how her child seemed more responsive to eating once she introduced a camera to document the activity. In showing off a picture of her smiling 9-year-old son with ASD Teo holding a plate of food, Paola said: “*Oh yes, he loves the camera, seeing the camera. So food became fun, because I was taking his picture when he ate. He did not complain at all. He even tried more food because I was taking his picture*.”

Parents with children with ASD also noted how the process of documenting food intake affected their children who were food selective to be slightly more open to trying new foods. Mother Susanna described her 6-year-old son Aiden as “a little less fussy” when she was taking pictures of him eating. She related: “*Normally he has his sensory thing where he won’t touch mushy food, like vegetables that have been cooked too long. But when I got out the camera, he thought it would be funny to pose with my plate and put his fork into the mashed potatoes, because he loves attention and being funny. I mean, he did not eat the mushy food … but at least he was playing with it, you know*?” Mother Estefania discussed her 8-year-old son with ASD Cristobal, as a picky eater who usually eats the same thing “meat and rice everyday”, but also how the camera served as a distraction to reduce his food selectivity. Estefania said: “*He only likes certain flavors and textures. But he saw the camera and me taking pictures of his food, and let me put some salad on his plate. I think it’s because he was more caught up with the camera being there that he wasn’t so resistant about what I served him*.”

Additionally, the research team also completed a table to record the types of foods documented in the photo journals. Notably, the foods that were documented the most were juice (appearing in photos from *n* = 13 children), pizza (appearing in photos from *n* = 12 children), and McDonald’s (appearing in photos from *n* = 13 children). These items were evenly distributed across the sample, appearing in photos from both TD children and children with ASD with similar ratios. Conversely, foods that were disproportionately skewed across groups were watermelon (*n* = 4; *n* = 3 TD and *n* = 1 ASD), water (*n* = 8; *n* = 1 ASD and *n* = 7 TD), fries (*n* = 9; *n* = 7 ASD and *n* = 2 TD), soda (*n* = 8; *n* = 7 ASD and *n* = 1 TD), cookies *n* = 5; *n* = 5 ASD and *n* = 0 TD), and milk (*n* = 4; *n* = 4 ASD and *n* = 0 TD).

## 4. Discussion

This qualitative visual methodology study examining diet and nutrition practices in Latinx families provides further details about food choices, food selectivity, and sugar consumption in an understudied group of children, and creates a rich picture of factors contributing to oral health status among Latinx children with and without ASD. The process of taking photos helped Latinx parents to better situate the barriers, beliefs, and behaviors influencing diet and food consumption in their children within the larger context of relating to their overall oral health. Additionally, parents became more attuned to the eating and food habits of their children, with more awareness of the daily activities of and food preferences of their families.

The first finding of this study was how the process of photo journaling empowered parents to be more aware of what their children ate, and the relationship of food choices to oral health. Indeed, every theme was related to what parents realized about their food habits as a result of participating in the research study. The benefit of involving the family in the research process was observing how the power dynamic changes, and inspires parents to be agentic, and make inferences about their children’s diet and oral health based on their observations of the photos. To this point, every parent commented about what they learned from the process of documenting their child’s food intake. Previous research with Latinx parents and caregivers has used a focus group methodology, and explored parents’ perceptions of a healthy lifestyle [43]. Ross and colleagues (2018) concluded that parent-identified barriers to healthy living included obstacles beyond their control, such as limited knowledge and education on health-promoting strategies, and difficulty with behavior change due to being used to a certain lifestyle. In this study, participants identified the barriers happening *to* them, and how they hoped to take actions in order to achieve better future outcomes. In cases where families faced systematic barriers like high costs of foods, parents were still able to note strategies they could employ in their given situation, such as reducing sugar intake before bed, to impact oral health outcomes. Participatory research, like the methods used in the present study, help move participants out of feeling powerless and toward authentic engagement and participation in their own health. Having parents who are motivated to actively participate in the care systems around them is significant, as parents of children with ASD are noted to be invaluable partners in informing health care providers about the unique dental care needs of their children [44,45]. By placing the camera in the hands of our participants and discussing the findings with them, our study empowered participants to recognize the multiple factors (e.g., institutional, community, interpersonal, and intrapersonal) that impact their diet-related health behaviors and decisions, and how to make impactful changes within their own means.

The next key significant finding of this photo journaling process was parents realizing the nutritional aspect of what their child ate, including how much sugar their child consumed, and how that related to oral health outcomes. The World Health Organization (WHO) has issued guidelines that recommend intake of free sugars (highly sugary products) to provide less than 10% of total food intake daily, and suggest further reductions to less than 5% of diet content to protect dental health throughout life [6]. In this study, some parents realized that their child had a preference for snacks that were sugary, and chose to consume foods that were less nutrient rich and more sugar filled, like a chocolate-covered cereal instead of a whole-grain based cereal, or having soda or a drink from Starbucks. They also realized the impact that consuming too much sugar may have on their child’s oral health, including causing caries. While multiple factors, such as levels of bacteria, saliva, and fluoride in the mouth, affect demineralization (disease) or remineralization (health) of tooth enamel during the caries development process, sugar consumption is especially precarious [46]. When sugar is consumed at high frequency and in a form that is retained in the mouth for long periods (such as in a bottle or juice), it is especially potent [47]. Sugar is cariogenic, because it allows for more bacterial adhesion while blocking the pH balancing effect of saliva [47]. Some families also recognized that they should try to limit sugary foods before bedtime, which is important to reducing caries development in their children [48], or mix water in with their children’s juice to serve their children less sugar.

Lastly, in line with previous research on food selectivity among children with disabilities including ASD [24,49], we anticipated that the food journaling process would indicate that the children with ASD were much more selective regarding food choices than the neurotypical children. Indeed, our findings show significant differences between frequency and types of certain foods that were consumed by children with ASD compared to the TD children, and differences in what the children with ASD *did not drink.* Children with ASD have significantly more feeding problems and eat a narrower range of foods than children without ASD [23,24]. Previous research has noted that as compared to TD children, children with ASD consume fewer raw vegetables, less fish and eggs, fewer legumes, citrus fruits, and more meat [50]. In our study, both children with ASD and TD children consumed an excess of sugar in the form of juice (*n* = 13 of 18 children), but children with ASD consumed even a greater amount of sugar than their peers because of the disproportionate ratio by which they consumed sweets. For example, of the eight children noted as having soda appear in their food journals, seven of those children has ASD. Similarly, of the five children who had cookies in their journals, all five came from the ASD group. A noteworthy observation is what was *missing* from photos from the ASD group, in that, of the eight children documented as drinking water, only *one* of them was from the ASD group; the remaining seven were from the TD group. Comparatively, this means that 87.5% of the TD group drank water, while only 10% of the ASD group did. Granted, as the journals were not completely comprehensive, is that the appearance of a photo in the food journal does not provide conclusive evidence that it was eaten; similarly, photos of food and drink that were consumed could be missing from the journals. However, it is a unique finding of our research that so few children with ASD were documented to drink water, and aligns with previous research about children with ASD consuming more sugary food items.

The finding that parents of both children with ASD and TD children mentioned pickiness in their children is also noteworthy; in our study, food selectivity was not solely observed in the ASD group. However, families of children with ASD did make some comments about their child’s sensory sensitivities making it difficult to introduce certain foods into the meal time routine, or how the presence of the camera sometimes increased their child’s willingness to be served different foods. Notably, parents of children with ASD did sometimes reference how their child “ate the same thing”, or had “sensory things” that made them completely avoid certain foods.

### Strengths and Limitations

Major strengths of this study included collecting rich, detailed information on perspectives of Latinx parents of children with and without ASD regarding their diet and food consumption practices. However, the current findings are not likely generalizable to all Latinx parents and caregivers of children with and without ASD, and should only be interpreted as expanding the body of current research. Moreover, aside from confirming a previous clinical diagnosis from a licensed profession, no diagnostic assessments were performed the children with ASD, nor were any intelligence or communication scales used. Therefore, all reporting on characteristics on the ASD group are from parent report and participant observations by the research team. Moreover, as time has passed since the participants were originally interviewed, these opinions are no longer the most current, and opinions or situations could have changed over this time period.

Additionally, given the self-expressive nature of the visual photo food journal, the type and quality of the photos taken across participants varied greatly. Notably, the families took photos of what their children were served, and not necessarily what was actually consumed by the children. Moreover, there were no questionnaires given to the families to describe meal components. Thus, noting the exact types of foods consumed, food preferences, and habits of the child was not measured nor standardized. Future research on diet can incorporate a form, such as a food frequency questionnaire or food recall form, that documents food consumption, food preparation information, environment where food was obtained, and amount of food consumed on each day. It would also be relevant to further the work on diet choices between neurotypical children and children with ASD and other developmental disabilities such as that explored by Bandini and colleagues [23,51].

## 5. Conclusions

The Latinx population has many unmet oral health needs deserving of more research to improve their health. The food selections made by Latinx parents and caregivers for their children or by the children, and the factors that impact these choices, including personal preference and systematic barriers, were analyzed using food journals and photo elicitation interviews. Visual research methodologies used in this study, such as photo journaling and photo elicitation interviewing, provide researchers with information that is authentic to the lived experiences of those providing the data [52], and involves the participants as part of the project. Using photo journaling methods of documenting food choices in picture form to connect occupational engagement to the experience of in-home oral care, daily occupations become opportunities to explore meaning-making and values [53,54]. Taking photos helped participants discuss aspects of their daily life, develop their awareness and perceptions of the situation surrounding their diet and oral health, and come to new conclusions about their routines based on their observations.

For example, the photo journaling process that we used helped parents to be more aware of both the positive and negative aspects of their child’s eating habits, the harmful impact of an overly sugary diet on oral care outcomes, and ways to encourage their child with ASD to try new foods and address food selectivity. Another important takeaway is about the food choices of the children themselves. While Latinx parents noted both their TD children and children with ASD as “picky” eaters, this study furthers the literature on food selectivity in ASD by showing how Latinx children with ASD consume more sugar and drink less water than their neurotypical peers. This finding has clear implications on oral health outcomes in Latinx children with ASD due to the negative impact of sugar consumption on caries development, and is an area that merits further research to create targeted education or intervention programs to reduce caries risk.

By introducing a visual methodology to the Latinx family dynamic, this study provided an opportunity to explore how this medically underserved population engages in the research process. Via their active participation in the research, parents were empowered to note strategies that they could employ that would directly impact their child’s oral health outcomes, such as reducing juice intake and monitoring sugar consumption. Therefore, visual research methodologies are an important strategy for researchers to consider in order to empower participants to be part of the research process and part of the outcomes. Particularly, visual methods, like photo journals, could be helpful in better understanding the lived experience of populations underrepresented in the literature, such as Latinx children with and without ASD and their families.

## Figures and Tables

**Table 1:** Sample Interview Questions.

Guiding Question
Tell me what your typical meals look like?
Who is involved in preparing meals in the home?
Where are your favorite places to eat in the community, and how frequently do you visit those places?
Do you feel your community has everything you need in it? (ex—grocery stores)
What aspects, if any, about dining and meals are challenging?
What foods do your child eat?
Does your child preferring eating the same thing every day?
What types of foods do you think are best for you to eat?
What types of foods do you think are not so good for you to eat?
Is there anything else we should know about your feelings and experiences related to this process?

**Table 2:** Participant Demographics (Participants are identified by pseudonyms to protect anonymity).

Name	Relationship to Child	Child’s Name	Child’s Age	ASD or TD Group	Language Preferred	Latino Cultural Background
Jennifer	Mother	Luca	7	ASD	English	Central American
Violeta	Mother	Justin	12	ASD	English	Mexican
Sara	Mother	Marco	8	ASD	English	Mexican
Isabella	Mother	Zach	8	ASD	English	Central American
Valeria	Mother	Juan	11	ASD	English	Mexican
Estefania	Mother	Cristobal	8	ASD	Spanish	Mexican
Armando	Father	Perlita	7	ASD	Spanish	South American
Paola	Mother	Teo	9	ASD	Spanish	Mexican
Susana	Mother	Aiden	6	ASD	English	Central American
Camila	Mother	Stefan	12	ASD	English	Mexican
Araceli	Mother	Julia	7	TD	Spanish	Mexican
Mia	Mother	Xochitl	11	TD	Spanish	Central American
Valentina	Mother	Ximena	11	TD	Spanish	South American
Celia	Mother	Ana Sofia	7	TD	Spanish	Mexican
Elisa	Mother	Miguel	12	TD	English	Central American
Carla	Mother	Samantha	8	TD	English	Central American
Maria Fernanda	Mother	Esperanza	6	TD	English	Mexican
Adriana	Mother	Christina	10	TD	English	Central American

## Data Availability

De-identified transcript data can be requested from the first author upon request and approval from the university review board.
